# The transcription factor *OpWRKY2* positively regulates the biosynthesis of the anticancer drug camptothecin in *Ophiorrhiza pumila*

**DOI:** 10.1038/s41438-020-00437-3

**Published:** 2021-01-01

**Authors:** Xiaolong Hao, Chenhong Xie, Qingyan Ruan, Xichen Zhang, Chao Wu, Bing Han, Jun Qian, Wei Zhou, Hans-Wilhelm Nützmann, Guoyin Kai

**Affiliations:** 1 Laboratory of Medicinal Plant Biotechnology, College of Pharmacy, Zhejiang Chinese Medical University, 310053 Hangzhou, China; 2 Institute of Plant Biotechnology, School of Life Sciences, Shanghai Normal University, 200234 Shanghai, China; 3 The Milner Centre for Evolution, Department of Biology and Biochemistry, University of Bath, Claverton Down, Bath, BA2 7AY UK

**Keywords:** Secondary metabolism, Metabolic engineering, Molecular engineering in plants, Metabolic engineering, Metabolic engineering

## Abstract

The limited bioavailability of plant-derived natural products with anticancer activity poses major challenges to the pharmaceutical industry. An example of this is camptothecin, a monoterpene indole alkaloid with potent anticancer activity that is extracted at very low concentrations from woody plants. Recently, camptothecin biosynthesis has been shown to become biotechnologically amenable in hairy-root systems of the natural producer *Ophiorrhiza pumila*. Here, time-course expression and metabolite analyses were performed to identify novel transcriptional regulators of camptothecin biosynthesis in *O. pumila*. It is shown here that camptothecin production increased over cultivation time and that the expression pattern of the WRKY transcription factor encoding gene *OpWRKY2* is closely correlated with camptothecin accumulation. Overexpression of *OpWRKY2* led to a more than three-fold increase in camptothecin levels. Accordingly, silencing of *OpWRKY2* correlated with decreased camptothecin levels in the plant. Further detailed molecular characterization by electrophoretic mobility shift, yeast one-hybrid and dual-luciferase assays showed that OpWRKY2 directly binds and activates the central camptothecin pathway gene *OpTDC*. Taken together, the results of this study demonstrate that OpWRKY2 acts as a direct positive regulator of camptothecin biosynthesis. As such, a feasible strategy for the over-accumulation of camptothecin in a biotechnologically amenable system is presented.

## Introduction

Camptothecin (CPT) is a monoterpenoid indole alkaloid (MIA) originally isolated from Chinese happy tree (*Camptotheca acuminata*)^1^. This natural product exhibits potent antitumor activity by inhibiting DNA topoisomerase I^1–3^. Its two derivatives, namely, topotecan and irinotecan, have been approved by the Food and Drug Administration (FDA) of the United States for the treatment of various cancers^4^. Due to the wide range of targets of CPT derivatives, their clinical demand is continuously increasing. Currently, commercial exploitation of camptothecin largely depends on extraction from natural woody plant resources such as *C. acuminata* and *Nothapodytes foetida*^4^. However, these plants typically show low camptothecin concentrations and are not suitable for modern biotechnological applications due to their long growth cycles. Several attempts have been made to establish robust plant cell culture systems to provide a platform for high CPT production. Among them are hairy-root cultures of *Ophiorrhiza pumila*, an herbal plant of the *Ophiorrhiza* genus in the *Rubiaceae* family. *O. pumila* produces camptothecin naturally in various organs, such as roots, stems, and leaves^5^. The *O. pumila* hairy-root system has been shown to produce up to 0.1–0.2% dry weight of camptothecin^6–8^. However, to further enhance camptothecin production in *O. pumila* by biotechnological engineering, it is crucial to fully dissect its biosynthetic pathway and molecular regulatory mechanism.

**Fig. 1:**
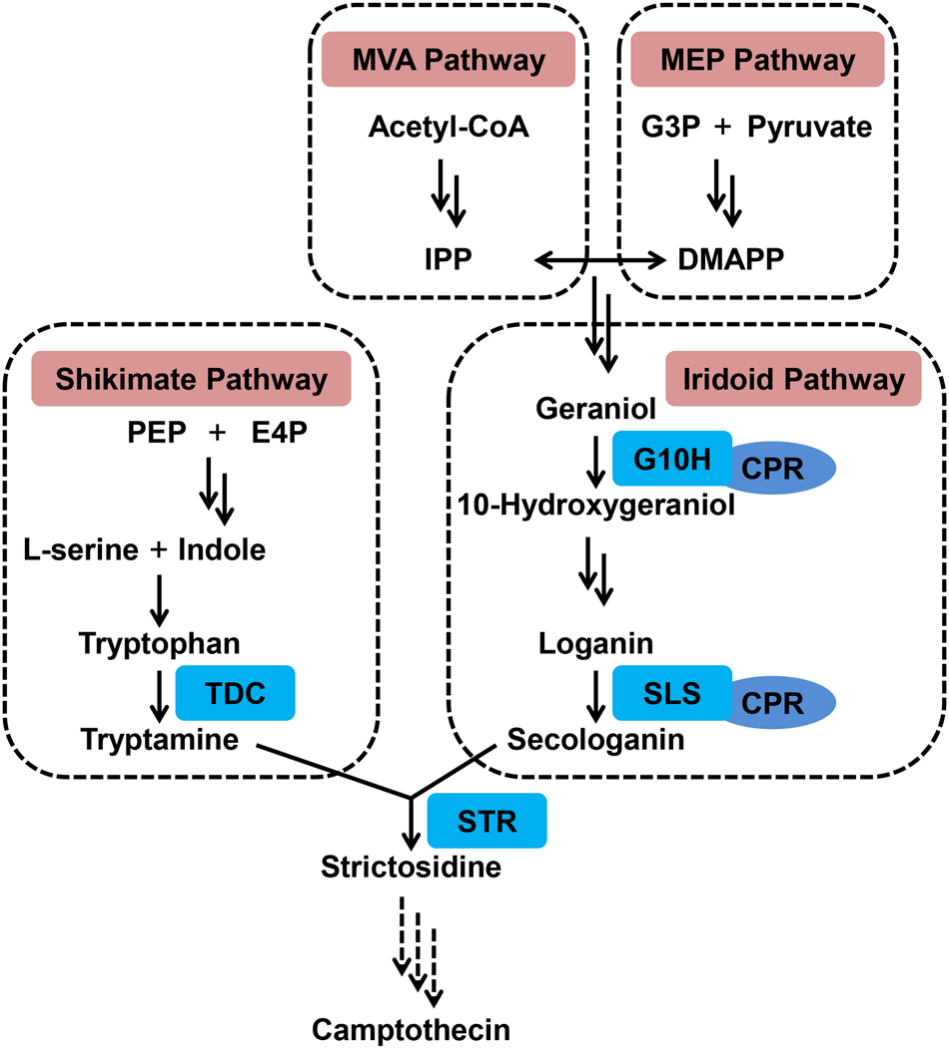
Camptothecin biosynthetic pathway in *O*. *pumila*. MVA pathway mevalonate pathway, MEP pathway 2-*C*-methyl-D-erythritol 4-phosphate pathway, IPP isopentenyl diphosphate, DMAPP dimethylallyl diphosphate, G10H geraniol-10-hydroxylase, SLS secologanin synthase, CPR cytochrome P450 reductase, TDC tryptophan decarboxylase, STR strictosidine synthase. Three dashed arrows indicate that the biosynthetic pathway is unknown.

The camptothecin biosynthesis pathway is complex and only partly resolved. Camptothecin biosynthesis uses intermediates of the iridoid and shikimate pathways, which converge to form the molecule strictosidine and then further, via unknown chemical reactions, form camptothecin (Fig. 1)^4,9^. Strictosidine synthase (OpSTR) catalyzes the condensation of tryptamine and secologanin to form strictosidine, a core intermediate in the camptothecin biosynthesis pathway^10^. Geraniol-10-hydroxylase (OpG10H) and secologanin synthase (OpSLS), two cytochrome P450 (CYP450) family synthases, are involved in the conversion of geraniol to secologanin in the iridoid pathway^8^. The cytochrome P450 reductase OpCPR is essential for the activity of OpG10H and OpSLS and plays an important role in electron transfer from nicotinamide adenine dinucleotide phosphate (NADPH) to cytochrome P450^8,10^. Tryptophan decarboxylase (OpTDC) catalyzes the conversion of tryptophan to tryptamine in the shikimate pathway^5,10^. Recent studies have confirmed that camptothecin biosynthesis can be manipulated by targeting these enzymes in metabolic engineering of *O. pumila* hairy roots. Suppression of *OpSLS* and *OpTDC* led to a decrease in camptothecin production in *O. pumila* hairy roots, thus corroborating their importance in camptothecin biosynthesis^11^. Manipulation of transgenic hairy-root systems further highlighted the important roles of both *OpG10H* and *OpSLS* in camptothecin biosynthesis, as co-overexpression of *OpG10H* and *OpSLS* increased camptothecin levels in *O. pumila* hairy roots^8^. However, less is known about the molecular regulatory mechanism of camptothecin biosynthesis in *O. pumila*.

WRKY transcription factors (TFs) are one of the largest transcriptional regulator families in plants. They characteristically contain one or two WRKY domains. These domains consist of ~60 amino acid residues and enable DNA binding^12–14^. The WRKY domain contains a highly conserved WRKYGQK amino acid sequence at its N-terminus and an atypical zinc-finger structure (C_2_H_2_ or C_2_HC) at its C-terminus^12,13,15^. In plants, WRKY TFs are divided into three groups based on the number of WRKY domains (two WRKY domains in Group I and one in Group II and III) and the structure of the zinc fingers (C_2_H_2_ in Group I and II and C_2_HC in Group III proteins)^12,13^. The WRKY domains generally recognize and bind to W-box (TTGACT/C) DNA motifs at target regions. Previous studies have shown that WRKY proteins, especially WRKY III subgroup members, are involved in the regulation of biosynthesis and accumulation of plant secondary metabolites in medicinal plants. For example, CrWRKY1, a WRKY III subgroup TF, has a positive regulatory effect on the biosynthesis of monoterpenoid indole alkaloids by binding and activating *CrTDC* in *Catharanthus roseus*^16^. In *Artemisia annua*, the WRKY III subgroup protein AaWRKY1 was reported to positively regulate artemisinin biosynthesis by promoting the transcription of *AaADS*, *AaCYP71AV1* and *AaDBR2*^17^. In *Salvia miltiorrhiza*, the WRKY III subgroup member SmWRKY1 was shown to positively regulate tanshinone biosynthesis by activating the *SmDXR* gene^15^. WsWRKY1, a WRKY III subgroup TF in *Withania somnifera*, was shown to positively regulate the biosynthesis of triterpenoids by activating *WsSQS* and *WsSQE*^18^. Recently, two WRKY III subgroup TFs, namely, OpWRKY1 and OpWRKY3, have been implicated in camptothecin biosynthesis in *O. pumila* hairy roots^5,19^. OpWRKY1 negatively regulates camptothecin biosynthesis, and OpWRKY3 plays a minor regulatory role in camptothecin production by affecting the development of *O. pumila* hairy roots and activating the expression of *OpCPR*^5,19^. In addition, several TFs from other families have also been reported to be involved in the regulation of camptothecin biosynthesis. OpERF2, an ERF TF family protein, has been isolated and shown to play a positive role in regulating camptothecin biosynthesis^20^. Moreover, introduction of the MYB TF member OpMYB1 into *O. pumila* hairy roots reduced camptothecin production^21^. In *C. acuminata*, a bZIP transcription factor, CaLMF, is a significant light signaling component and mediates light-regulated camptothecin biosynthesis^22^. In general, transcriptional regulation plays an important role in the biosynthesis of bioactive metabolites in medicinal plants.

In this study, the biosynthesis of camptothecin in *O. pumila* hairy roots at different growth stages was systematically investigated. By analyzing the expression of all putative WRKY TFs of *O. pumila* at different growth stages, a single transcription factor, *OpWRKY2*, was identified as being highly coexpressed with known camptothecin biosynthesis pathway genes. Overexpression of *OpWRKY2* in *O. pumila* hairy roots resulted in increased levels of *OpTDC* expression as well as camptothecin and tryptamine formation. Accordingly, downregulation of *OpWRKY2* reduced *OpTDC* expression levels and camptothecin production. Biochemical characterization showed that OpWRKY2 binds and activates the promoter of *OpTDC* in vitro and in vivo. Altogether, our analyses identified a novel regulator of camptothecin biosynthesis and a potential target for advanced bioengineering for enhanced production of an important anticancer drug.

## Materials and methods

### Plant materials

In this study, the *O. pumila* plants originated from Fujian Province of China and were cultivated in a greenhouse at Zhejiang Chinese Medical University. The *O. pumila* plantlets used for analysis of tissue expression patterns and hairy-root transformation were obtained and cultured in solid B5 medium as previously reported^7^. Three different tissues of two-month-old *O. pumila* sterile plants (roots, stems and leaves) were separately collected for tissue expression pattern analyses of *OpWRKY2* and *OpTDC* and camptothecin content detection in different tissues. Different *O. pumila* hairy-root lines were inoculated and cultured in liquid B5 medium at 120 rpm in darkness at 25 °C. *Nicotiana benthamiana* plants used for subcellular localization analyses of OpWRKY2 protein and dual-luciferase (Dual-LUC) assays to detect the activation of OpWRKY2 were grown in pots containing soil mixture and placed in a growth chamber under a light/dark photoperiod of 16/8 h at 25 °C^23^.

### Hairy roots at different growth stages and phytohormone treatments

For analysis of growth phenotype and camptothecin production in *O. pumila* hairy roots, 0.2 g of fresh C58C1 hairy-root lines (infected with modified *Agrobacterium tumefaciens* strain C58C1) with uniform growth were inoculated in B5 liquid medium and cultured for 60 days^7,15^. Hairy roots and culture medium were harvested at 10, 20, 30, 40, 50, and 60 days and used to detect gene expression and metabolite levels. For the analysis of *OpWRKY2* gene expression in response to plant hormones, C58C1 hairy-root lines were cultured for 30 days and subsequently treated with 50 μM salicylic acid (SA), 50 μM gibberellin (GA_3_), 100 μM abscisic acid (ABA), and 200 μM methyl jasmonate (MeJA)^5^. An equivalent volume of ethanol was used as a control. The hairy roots were harvested at 0, 0.5, 1, 3, 6, 9, and 12 h post hormone application and immediately frozen in liquid nitrogen for further quantitative real-time polymerase chain reaction (qRT-PCR) analyses.

### Plant RNA extraction and qRT-PCR analyses

Total RNA of all samples was extracted using the Plant RNAprep Pure Kit (Tiangen, Beijing, China). cDNA synthesis from total RNA and qRT-PCR analyses of all gene transcripts were performed as previously described^24^. The housekeeping gene *OpActin* in *O. pumila* was used as an internal control gene in qRT-PCR for normalization of all samples. All gene-specific primer sequences of all camptothecin biosynthesis pathway genes and *OpWRKY* genes used for qRT-PCR analyses are listed in Table S1. The relative gene expression values in all samples were calculated using the 2^−ΔΔCt^ method. All qRT-PCR analyses of each sample were performed for three biological replicates.

### Bioinformatics analysis

The bioinformatics analyses were performed as previously described^25^. WRKY homologues were identified in the *O. pumila* root and hairy-root transcriptome sequences generated by our laboratory using the hidden Markov model (HMM) search and BLASTx program with an *E-*value < 10^−5^ ^8,25^. Redundant sequences were manually removed. All OpWRKY proteins in *O. pumila* were aligned using the ClustalW program with the default parameters^26^. The phylogenetic tree of all OpWRKY proteins in *O. pumila* was constructed and performed using the neighbor-joining method with MEGA5 software^27^. The bootstrap values were calculated from 1000 replicates to analyze and assess the accuracy of the phylogeny.

### Subcellular localization

To analyze the subcellular localization of the OpWRKY2 protein, the open reading frame (ORF) was amplified by PCR from the *O. pumila* hairy-root cDNA library with *OpWRKY2* gene-specific primers (Table S2) and inserted into the modified plant expression vector *pHB-YFP* (yellow fluorescent protein) to generate the *pHB-OpWRKY2-YFP* construct. The *pHB-YFP* construct without *OpWRKY2* was used as the negative control. The plasmids *pHB-OpWRKY2-YFP* and *pHB-YFP* were introduced into the *A. tumefaciens* strain GV3101 and transiently infected the epidermal cells of 5-week-old *N*. *benthamiana* leaves. YFP signals were analyzed using an LSM880 confocal laser microscope (Carl Zeiss, Germany) 48 h post infection, and three biological replicates were performed to confirm the results as reported previously^23,28^.

### Generation of *OpWRKY2* transgenic *O. pumila* hairy roots

The full-length *OpWRKY2* ORF fragment was amplified with *Spe* I and *BstE* II restriction sites and inserted into the previously modified *pCAMBIA2300*^*+*^ plant expression vector to obtain the *pCAMBIA2300*^*+*^*-OpWRKY2* construct, which was used for overexpression of *OpWRKY2* in *O. pumila* hairy roots (Fig. S1a)^7,15^. Chimeric repressor silencing by using the EAR motif repression domain (SRDX) is a very common technique that is widely used to study the function of transcription factors^29^. It is very useful not only for the rapid analysis of the function of redundant transcription factors but also for the manipulation of biological traits via the suppression of gene expression that is regulated by specific transcription factors^29^. In this study, to further examine the function of *OpWRKY2*, the DNA sequence encoding the SRDX repressor domain (LDLDLELRLGFA) was fused to the C-terminus of *OpWRKY2* and inserted into the modified *pCAMBIA2300*^*+*^ to generate the *pCAMBIA2300*^*+*^*-OpWRKY2-SRDX* construct (Fig. S1b)^16,29^. The *pCAMBIA2300*^*+*^ empty vector without *OpWRKY2* was used as the control. All plasmids were transferred into the disarmed *A. tumefaciens* strain C58C1, harboring the *Agrobacterium rhizogenes* Ri plasmid pRiA4, and subsequently transformed into *O. pumila* stems to generate *O. pumila* transgenic hairy-root lines^7,15^. The screening of transformed hairy-root cultures was carried out on B5 medium plates. The positive transgenic hairy-root lines (*OpWRKY2*-*OE* and *OpWRKY2*-*SRDX*) were verified via PCR amplification of hairy-root genomic DNA. Primers used for PCR amplification of positive transgenic hairy roots were designed to cover the *OpWRKY2* gene and partial *pCAMBIA2300*^*+*^ vector sequences and are listed in Table S2. Positive transgenic hairy roots were further inoculated and cultured in B5 liquid medium for 45 days in the dark. Harvested hairy-root lines were used for gene expression and metabolite analysis.

### Determination of metabolites in hairy roots by HPLC

For a time series of metabolite production in *O. pumila*, hairy roots were harvested every 10 days for 60 days. Hairy roots of transgenic lines, namely, *OpWRKY2-OE* and *OpWRKY2-SRDX*, were harvested after 45 days of cultivation. All samples were dried at 50 °C and thoroughly ground, and the liquid medium was directly evaporated. These samples were used for further metabolite measurements.

For camptothecin and tryptamine measurements, ~0.1 g of dried hairy-root powder was extracted with 20 mL of methanol and sonicated for 1 h. After centrifugation, the supernatant was evaporated under vacuum, and the residual material was dissolved in 2.0 mL of methanol and filtered through a 0.22 μm filter. High-performance liquid chromatography (HPLC) analysis of camptothecin and tryptamine extracts was performed using an Agilent 1260 detector equipped with a reversed-phase C18 column (Agilent Technologies, Palo Alto, CA, USA) as previously described^5,8^. The camptothecin detection conditions were as follows: mobile phase, acetonitrile:water (65:35, v/v); column temperature, 30 °C; and detection wavelength, 254 nm. Tryptamine detection conditions were as follows: mobile phase, 43% acetonitrile:30% methanol:26% double distilled water:1% glacial acetic acid (v/v); column temperature, 30 °C; detection wavelength, 254 nm. Commercially available standards of camptothecin and tryptamine (Aladdin, Shanghai, China) were used for identification and comparative quantification as previously described^5,8^.

For loganin and secologanin measurements, ~0.1 g of dried hairy-root powder was extracted with 10 mL of ethanol:water (4:1, v/v) and sonicated for 30 min. After centrifugation, the supernatant was evaporated under vacuum, and the residual material was dissolved in 2.0 mL of water and filtered through a 0.22 μm filter. HPLC analysis of loganin and secologanin extracts was performed using an Agilent 1260 detector equipped with a reversed-phase C18 column (Agilent Technologies, Palo Alto, CA, USA) as previously described^5,8^. The detection conditions of loganin and secologanin were as follows: mobile phase, acetonitrile:water (25:75, v/v); column temperature, 30 °C; and detection wavelength, 236 nm. Commercially available standards of loganin and secologanin (Aladdin, Shanghai, China) were used for comparative quantification as previously described^5,8^.

### Recombinant protein production and EMSA

To express and purify the recombinant protein, the full-length *OpWRKY2* ORF fragment was cloned into the *BamH* I and *Hind* III sites of the *pCold-TF* vector. The constructs were verified by DNA sequencing and transformed into *Escherichia coli* strain Rosetta (DE3) cells to produce His-tagged fusion proteins. The *pCold-TF* empty vector without *OpWRKY2* was used as the negative control. Transformed Rosetta cell cultures used for the expression of HIS recombinant protein were induced by adding isopropyl β-D-thiogalactopyranoside (IPTG) at a final concentration of 0.1 mM at an optical density of approximately 0.6 at 600 nm. After induction for 14 h at 16 °C, Rosetta cells were harvested by centrifugation and purified using Ni-NTA (nitrilotriacetic acid) agarose (Invitrogen, USA) as previously described^16^. To investigate the ability of the OpWRKY2 protein to bind to the W-box in the *OpTDC* promoter, the 3090 bp upstream region of the *OpTDC* gene was analyzed. For Electrophoretic mobility shift assay (EMSA) experiments, DNA probes were designed based on the native *OpTDC* promoter sequence (−2565 to −2552 relative to the ATG) containing a single W-box. Complementary oligonucleotides labeled with biotin at the 5’ end of each strand were synthesized and annealed to produce double-stranded probes for EMSA. EMSAs were performed as previously described^16^.

### Dual-LUC assays

To investigate the ability of OpWRKY2 to transcriptionally activate the *OpTDC* gene, the 3090 bp promoter of *OpTDC* was analyzed and cloned into the *pGreenII0800-LUC* vector. The reporter constructs *wt-pOpTDC*::*fLUC* and *mutant-pOpTDC*::*fLUC* were obtained by inserting the native promoter of *OpTDC* and a mutated version into the *pGreenII0800-LUC* vector to drive expression of the *firefly luciferase* gene^30^. The *Renilla luciferase* gene driven by the CaMV 35 S promoter was used as an internal control. The assembled vectors were cotransformed with the helper plasmid *pSoup19* into *A. tumefaciens* strain GV3101. The *A. tumefaciens* strain GV3101 containing *pHB-OpWRKY2-YFP* was used as the effector, and *pHB-YFP* was used as the negative control. Infiltration and detection were performed as previously described, with minor modifications^31^. The reporter strains harboring *wt-pOpTDC*::*fLUC* or *mutant-pOpTDC*::*fLUC* were mixed with effector strains harboring either *pHB-OpWRKY2-YFP* or *pHB-YFP* at a ratio of 1:1. Leaves were collected after 48 h, and Dual-LUC assays were performed using the Dual-Luciferase Reporter Assay System according to the manufacturer’s instructions (Promega, Madison, WI, USA). Three biological replicates per treatment were measured. All primers used to amplify the *OpTDC* promoter are listed in Table S2.

### Y1H assays

For Y1H assays, the full-length *OpWRKY2* ORF fragment was amplified and cloned into the effector plasmid *pB42AD*. The triple tandem copy of the *pOpTDC* wt-W-box motif (CTTC**AGTCAA**GGCC) and mutant-W-box motif (CTTC**AttttA**GGCC) were inserted into the reporter plasmid pLacZ between *EcoR* I and *Xho* I. Y1H assays were performed as previously described^23,32^. Effector and reporter plasmids were cotransformed into yeast strain EGY48a. Transformants were cultivated on SD/-Ura/-Trp medium for 48 h and tested on SD/-Ura/-Trp medium with 5-bromo-4-chloro-3-indolyl-β-D-galactopyranoside (X-gal) for 24 h. Empty *pB42AD* and *pLacZ* plasmids were cotransformed into yeast and used as negative controls. All primers used to amplify *OpWRKY2* and DNA motifs are listed in Table S2.

### Statistical analyses

All experiments in this study were conducted with at least three biological replicates. All data are presented as the mean ± standard deviation (SD). To test the statistically significant differences between the control and treated samples/transgenic hairy-root lines, a paired two-tailed Student’s *t*-test was conducted with a significance threshold of *p* < 0.05.

## Results

### Camptothecin biosynthesis at different growth stages of *O. pumila* hairy roots

**Fig. 2:**
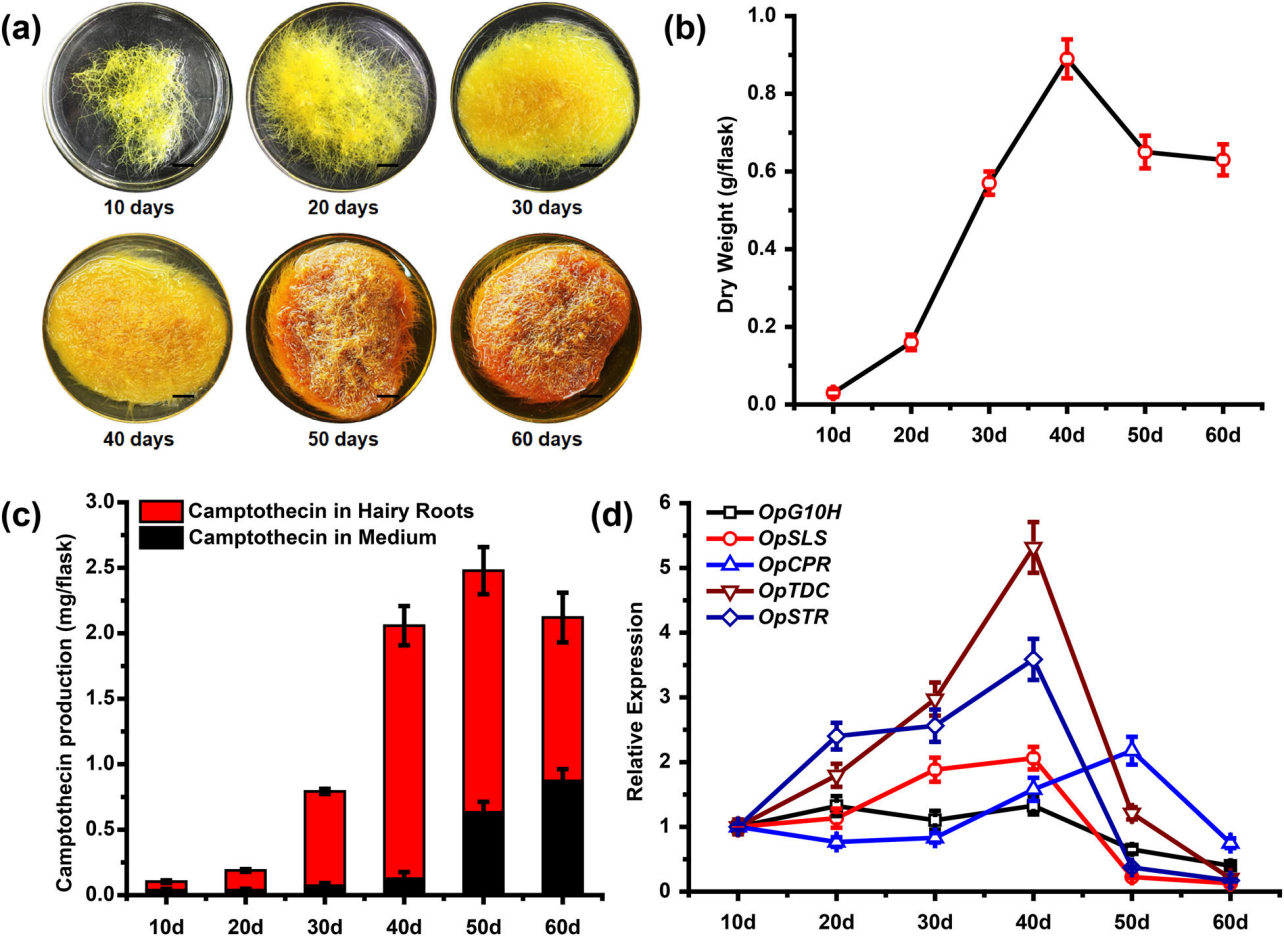
The growth phenotype and camptothecin biosynthesis ability of *O*. *pumila* hairy roots at different growth stages. **a** The growth phenotype of *O. pumila* hairy roots at different growth stages (scale bars: 1 cm). **b** The biomass of *O. pumila* hairy roots at different growth stages. Error bars represent the SD of three biological replicates. **c** The production of camptothecin in hairy roots and liquid medium was detected by HPLC. Error bars represent the SD of three biological replicates. **d** The transcription of five camptothecin biosynthetic genes at different growth stages was detected by qRT-PCR analyses. The transcriptional expression level of each gene at 10 days was set to 1. The *OpActin* gene was used as the internal reference gene. Error bars represent the SD of three technical replicates.

Hairy roots of *O. pumila* have the potential to synthesize camptothecin, yet the association of cultivation time and camptothecin production remains poorly characterized. To investigate the capacity of *O. pumila* hairy roots to produce camptothecin at different growth stages, ~0.2 g of fresh hairy roots with uniform growth were inoculated in B5 liquid medium and cultured for 60 days. As shown in Fig. 2a, b, the color and biomass of the hairy roots changed at different growth stages over time. The color of the hairy roots gradually intensified, changing from yellow to dark yellow, and the color of the medium changed from colorless to yellow (Fig. 2a). The biomass of the hairy roots gradually increased, reaching a maximum of 0.89 g dry weight at 40 days (Fig. 2b).

Since camptothecin in *O. pumila* hairy roots could be excreted into the culture medium, we analyzed hairy roots and liquid medium separately by HPLC analysis to monitor the accumulation of camptothecin in *O. pumila* (Fig. 2c). The total yield of camptothecin increased over time and reached a maximum of 2.48 mg/flask at 50 days (Fig. 2c). Interestingly, the content of camptothecin in the culture medium increased throughout the growth period and reached 0.87 mg/flask at 60 days. In contrast, the content of camptothecin extracted from hairy roots peaked at 40 days at ~1.93 mg/flask (Fig. 2c).

In parallel to the metabolite analyses, the expression levels of camptothecin biosynthetic genes (*OpG10H, OpSLS, OpCPR, OpTDC*, and *OpSTR*) were determined by qRT-PCR analyses. As shown in Fig. 2d, the relative transcript levels of all camptothecin biosynthetic genes increased over time. The highest upregulation was detected for the biosynthesis genes *OpTDC* and *OpSTR*, reaching maximum expression levels after 40 days (Fig. 2d). After 50 days, the relative expression levels of *OpG10H, OpSLS, OpTDC*, and *OpSTR* decreased (Fig. 2d). Expression of the *OpCPR* gene peaked at 50 days and decreased slightly at the final time point (Fig. 2d). Altogether, these data suggested that camptothecin biosynthesis in *O. pumila* hairy roots changes dynamically over time.

### Identification of expressed WRKY TFs in *O. pumila* hairy roots

**Fig. 3:**
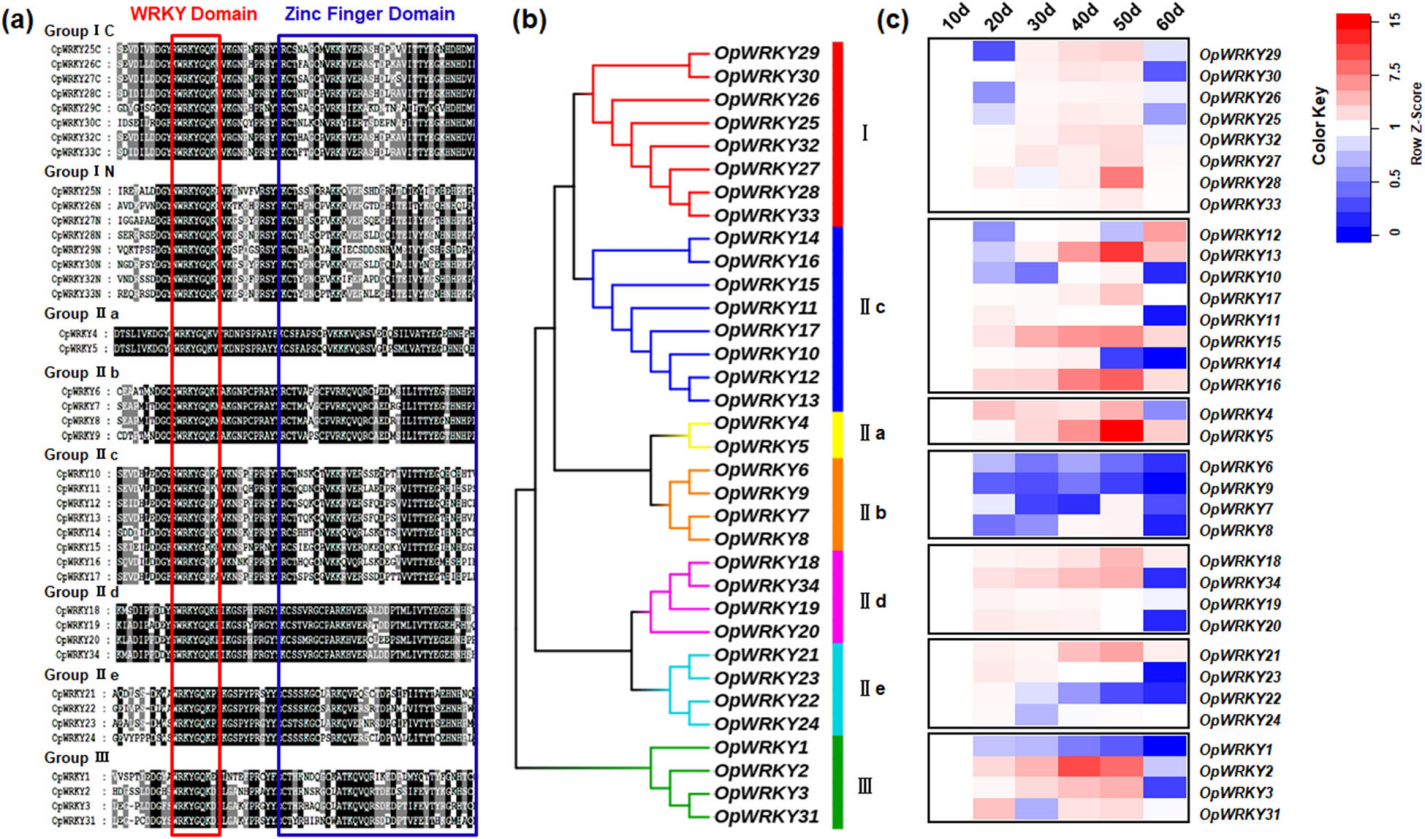
Sequence and expression analysis of OpWRKYs. **a** Protein sequence alignment of OpWRKYs. The conserved WRKY domains are highlighted in red; the conserved zinc-finger domains are highlighted in blue. **b** Phylogenetic analysis of OpWRKYs. **c** The expression levels of all *OpWRKY* genes at different growth stages were detected by qRT-PCR analyses. The expression level of each gene at 10 days was set to 1. The *OpActin* gene was used as the internal reference gene.

Previously, it has been reported that WRKY TFs are involved in the regulation of secondary metabolism in medicinal plants. To systematically investigate the importance of OpWRKYs in the positive regulation of camptothecin biosynthesis, an available *O. pumila* hairy-root transcriptome dataset was analyzed for expressed WRKY domain-encoding genes. Thirty-four putative WRKY transcription factor genes encoding either one or two WRKY domains were identified (Fig. 3a, b). Phylogenetic analysis based on the protein sequences divided the putative OpWRKY TFs into three groups (Fig. 3a, b). Protein alignments showed that 8 OpWRKYs fell into WRKY TF group I, 22 OpWRKYs to WRKY TF group II, and 4 OpWRKYs to WRKY TF group III (Fig. 3a).

To further characterize these WRKY TFs and to better understand their role in camptothecin biosynthesis, the relative expression levels of all putative WRKY TFs were determined by qRT-PCR at different growth stages by qRT-PCR (Fig. 3c). Variable expression trends were observed for the OpWRKY TF genes. The expression levels of a number of *OpWRKY* genes increased over time, while the expression levels of others gradually decreased. The expression pattern of only one OpWRKY TF gene, *OpWRKY2*, closely mirrored the pattern observed for camptothecin biosynthetic genes. *OpWRKY2* expression gradually increased until it reached a peak at 40 days, slightly decreased at 50 days and dropped to a very low level at 60 days (Fig. 3c). Taken together, these results indicated that *OpWRKY2* might be a candidate gene involved in the positive regulation of camptothecin biosynthesis.

### Expression profiling and subcellular localization of *OpWRKY2*

**Fig. 4:**
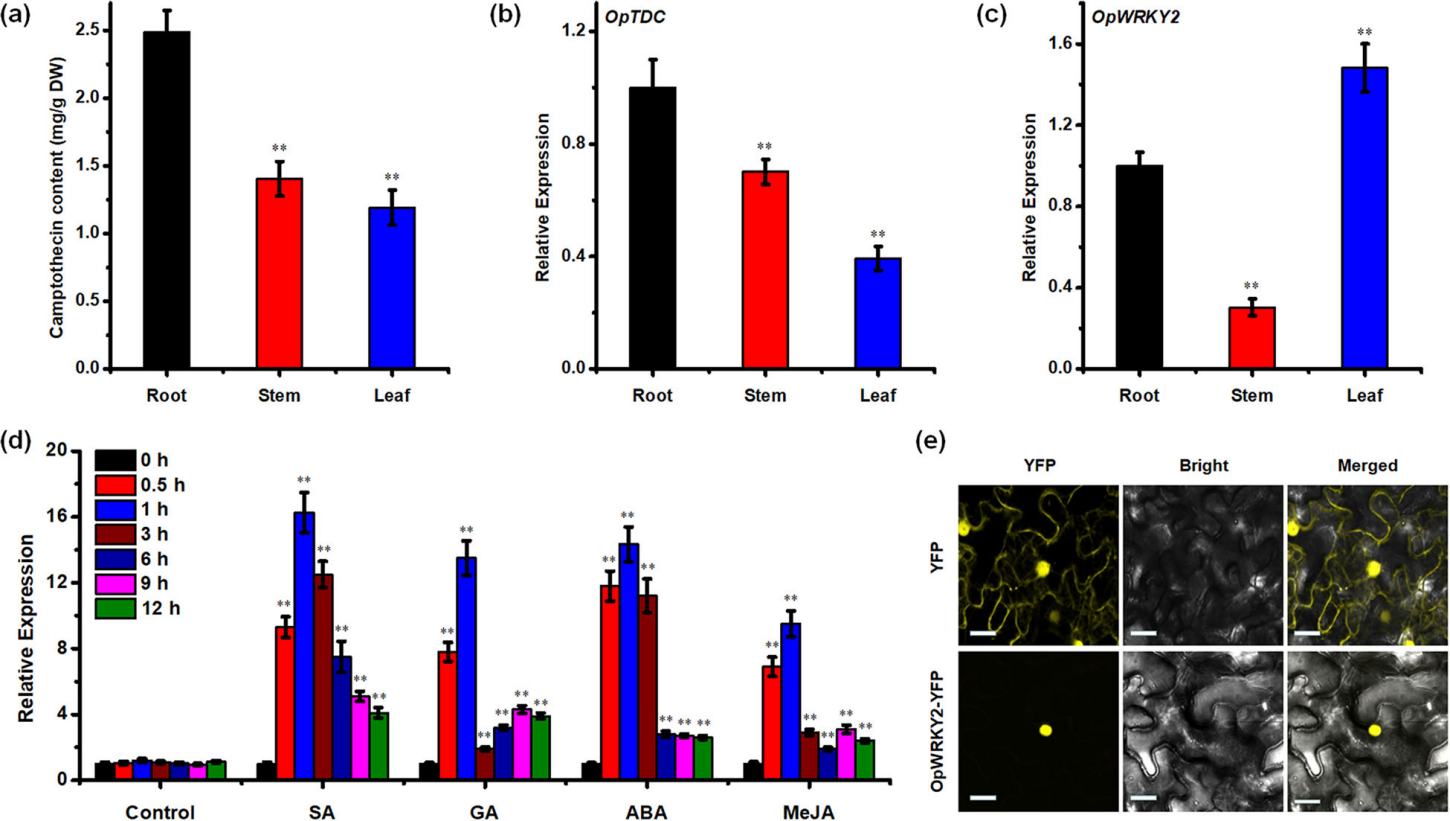
Expression patterns of *OpWRKY2*. **a** The production of camptothecin in the roots, stems, and leaves of *O. pumila* was detected by HPLC. Error bars represent the SD of three biological replicates. **b, c** The expression levels of *OpTDC* (**b**) and *OpWRKY2* (**c**) in the roots, stems, and leaves of *O. pumila* were measured by qRT-PCR. Gene expression levels in roots were set to 1. **d** The expression levels of *OpWRKY2* genes under different phytohormone treatments were detected by qRT-PCR analyses. The gene expression level at 0 h was set to 1. The *OpActin* gene was used as the internal reference gene. Error bars represent the SD of three technical replicates. **e** The subcellular localization of OpWRKY2. The subcellular localization of 35S::OpWRKY2-YFP and 35S::YFP in *N. benthamiana* leaf epidermal cells. Scale bars: 20 μm.

To further investigate the expression pattern of *OpWRKY2*, we collected materials from three different tissues (root, stem and leaf) of 2-month-old *O. pumila* plants and analyzed camptothecin biosynthesis and the relative transcript levels of *OpWRKY2*. First, we investigated the content of camptothecin in different tissues and found that the accumulation level of camptothecin in the roots, stems and leaves of *O. pumila* was quite high, with the highest content observed in roots (Fig. 4a). Then, we detected the expression of the camptothecin biosynthetic gene *OpTDC* in different tissues and found that it was expressed in roots, stems and leaves, and the expression level was relatively high in roots (Fig. 4b). In addition, the highest transcript levels of *OpWRKY2* were detected in leaves, intermediate levels in roots and low levels in stems (Fig. 4c). These results indicate that *OpWRKY2* might not only be involved in the regulation of camptothecin biosynthesis but also regulate other processes in plant growth and development.

Next, the transcriptional response of *OpWRKY2* to exogenous phytohormone treatments was analyzed. Thirty-day-old *O. pumila* hairy roots were individually treated with the plant hormones SA, GA_3_, ABA and MeJA, and samples were taken after 0, 0.5, 1, 3, 6, 9, and 12 h. The results presented in Fig. 4d show that application of each hormone led to rapid and significant upregulation of *OpWRKY2* transcript levels (Fig. 4d). In response to SA and ABA, *OpWRKY2* expression increased 14-fold after 1 h and was maintained at high levels for at least 3 h. In contrast, in response to GA and MeJA, *OpWRKY2* expression peaked within 1 h and returned to near basal levels at 3 h. These results indicate an important role of hormones in the transcriptional regulation of *OpWRKY2*.

To analyze the subcellular localization of OpWRKY2, we carried out transient transformation assays in *N*. *benthamiana* leaves. As shown in Fig. 4e, fluorescent *OpWRKY2*-reporter signals were specifically detected in the nucleus of *N*. *benthamiana* leaf cells, while the YFP control protein was distributed throughout the cells (Fig. 4e). This nuclear localization is in accordance with the expected role of OpWRKY2 as a transcription factor.

### Generation of the *OpWRKY2-OE* and *OpWRKY2-SRDX* hairy-root lines

**Fig. 5:**
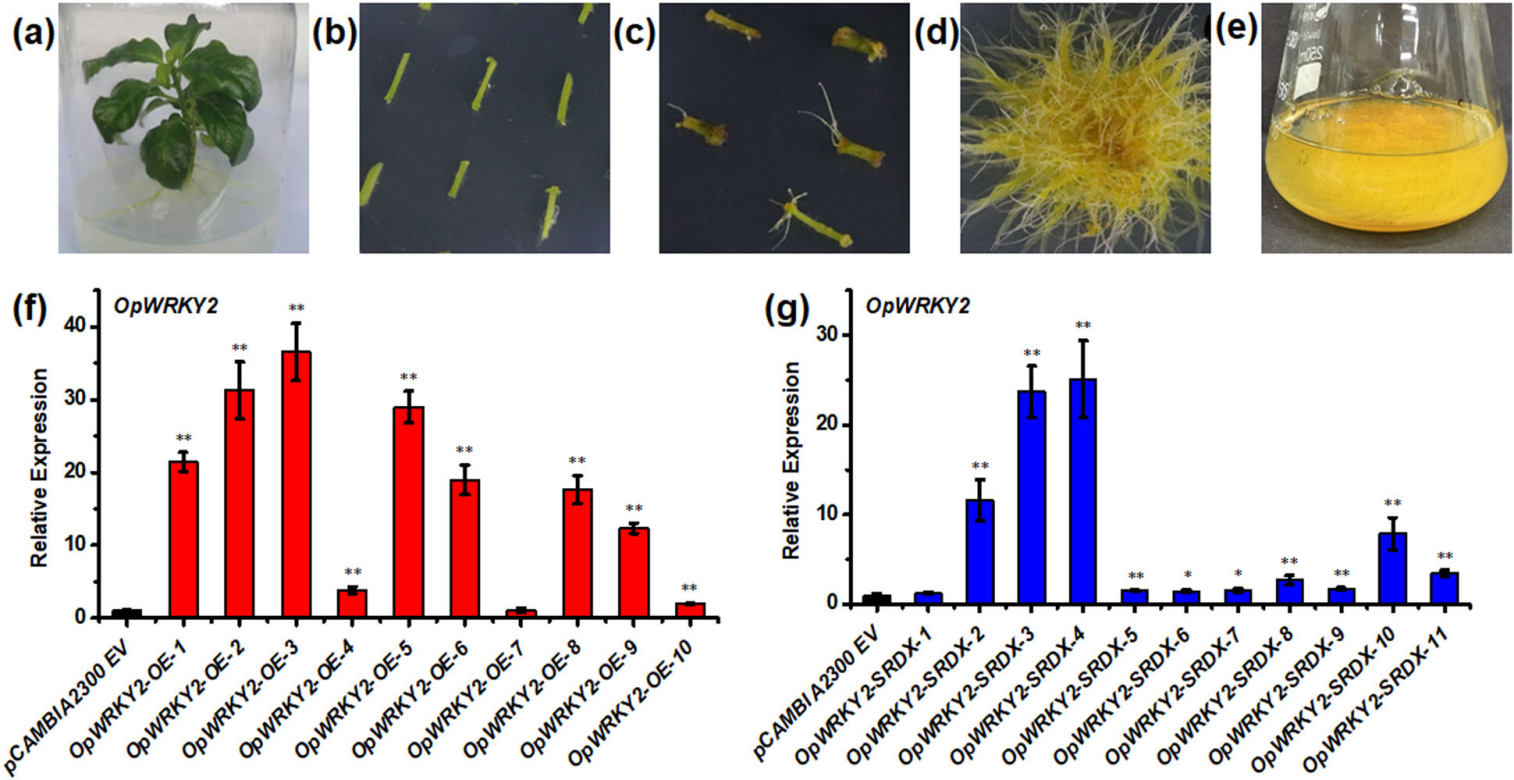
Generation of transgenic *O. pumila* hairy-root lines. **a** *O. pumila* explants on B5 medium. **b** *O. pumila* stems precultured on B5 medium. **c** Hairy roots differentiated from infected *S. miltiorrhiza* explants. **d** Isolated monoclonal hairy roots. **e** Hairy-root monoclones cultured in B5 liquid medium. **f, g** The relative transcript levels of *OpWRKY2* in the *OpWRKY2-OE* (**f**) and *OpWRKY2-SRDX* (**g**) transgenic hairy-root lines were detected by qRT-PCR. The *pCAMBIA2300*^*+*^ empty vector was used as a control. The average expression level of *OpWRKY2* in the two control hairy-root lines was set to 1. The *OpActin* gene was used as the internal reference gene. Error bars represent the SD of three technical replicates.

To establish a role for *OpWRKY2* in camptothecin biosynthesis, *OpWRKY2* overexpression and silencing lines were generated. For *O. pumila* hairy roots, the recombinant overexpression and silencing constructs were introduced to *O. pumila* explant stems by Agrobacterium-mediated transformation (Fig. 5a–e). Positive transgenic hairy-root lines carrying *OpWRKY2* were identified by PCR on genomic DNA of *O. pumila* using gene-specific primers (Table S2). The results showed that 17 out of 48 tested *OpWRKY2-OE* hairy roots were successfully transformed (35.4% positive rate) (Fig. S2). In addition, 15 out of 32 candidate *OpWRKY2-SRDX* hairy roots were successfully transformed (46.9% positive rate) (Fig. S3). Expression analysis of *OpWRKY2* in *OpWRKY2-OE* lines showed a 1.03- to 36.55-fold increase in relative transcript levels compared to the wild-type control (Fig. 5f). In *OpWRKY2-SRDX* hairy-root lines, relative transcript levels of *OpWRKY2* were increased 1.26- to 25.12-fold compared to the wild-type control (Fig. 5g). For both *OpWRKY2-OE* and *OpWRKY2-SRDX*, 4 lines with the highest increase in expression were selected for further analysis.

### Expression analysis of camptothecin biosynthesis genes in *OpWRKY2-OE* and *OpWRKY2-SRDX* hairy roots

**Fig. 6:**
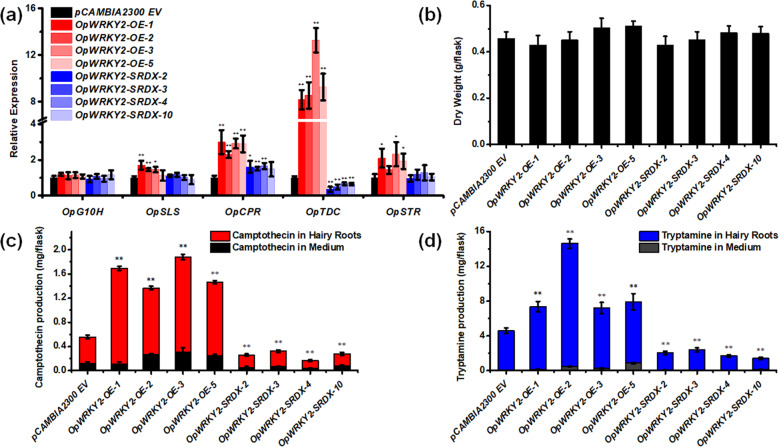
Analysis of camptothecin biosynthesis in the *OpWRKY2-OE* and *OpWRKY2-SRDX* transgenic hairy-root lines. **a** The expression levels of camptothecin biosynthetic genes in the *OpWRKY2-OE* and *OpWRKY2-SRDX* transgenic hairy-root lines. The average transcriptional expression level of each gene in the two control hairy-root lines was set to 1. The *OpActin* gene was used as the internal reference gene. Error bars represent the SD of three technical replicates. **b** The biomass of *OpWRKY2-OE* and *OpWRKY2-SRDX* transgenic hairy-root lines. Error bars represent the SD of three biological replicates. **c, d** The production of camptothecin (**c**) and tryptamine (**d**) in *OpWRKY2-OE* and *OpWRKY2-SRDX* transgenic hairy-root lines was detected by HPLC. Error bars represent the SD of three biological replicates.

To analyze the impact of *OpWRKY2* on the expression of camptothecin pathway genes, the relative transcript levels of *OpG10H, OpSLS, OpCPR, OpTDC*, and *OpSTR* were measured in *OpWRKY2* overexpression and silencing lines (Fig. 6a). The relative transcript levels of *OpTDC* were increased almost 10-fold in *OpWRKY2-OE* hairy-root lines compared to the control. Accordingly, *OpTDC* expression levels were significantly decreased in the *OpWRKY2-SRDX* hairy-root lines, suggesting a positive regulatory role of *OpWRKY2* in *OpTDC* expression. The relative expression of *OpG10H* and *OpSLS*, two CYP450 family genes in the iridoid pathway, did not change significantly in the *OpWRKY2-OE* and *OpWRKY2-SRDX* hairy-root lines. In addition, the expression levels of *OpCPR* and *OpSTR* were increased slightly in the *OpWRKY2-OE* hairy-root lines compared to the control, but there was no obvious change in the *OpWRKY2-SRDX* hairy-root lines. In conclusion, OpWRKY2 positively regulates the expression of *OpTDC* in *O. pumila* hairy roots.

### Metabolite analysis of *OpWRKY2-OE* and *OpWRKY2-SRDX* hairy roots

To analyze metabolite levels in the generated lines, hairy roots of *O. pumila* were inoculated into 100 mL of liquid B5 medium for 45 days and then collected for further study. Neither overexpression nor silencing of *OpWRKY2* significantly affected the dry weight of the analyzed hairy roots compared to the wild-type control (Fig. 6b). HPLC analysis of camptothecin levels in *OpWRKY2-OE* lines showed a 1.7–3.4-fold increase compared to the control (Fig. 6c), indicating that overexpression of *OpWRKY2* is effective in promoting the accumulation of camptothecin in *O. pumila*. Accordingly, the average content of camptothecin in the *OpWRKY2-SRDX* lines decreased slightly in comparison to the control lines (Fig. 6c). The levels of tryptamine, a key intermediate in the biosynthesis of camptothecin, increased 1.83-fold in *OpWRKY2*-overexpressing lines and decreased by approximately half in SRDX lines (Fig. 6d). In contrast, the levels of loganin and secologanin, which are pathway intermediates in the iridoid branch of camptothecin biosynthesis, showed high variability and no obvious trend in the *OpWRKY2-OE* and *OpWRKY2-SRDX* lines (Fig. S4). Altogether, these results suggest a positive regulatory role of *OpWRKY2* in camptothecin biosynthesis.

### OpWRKY2 activates the transcription of *OpTDC* in vitro and in vivo

**Fig. 7:**
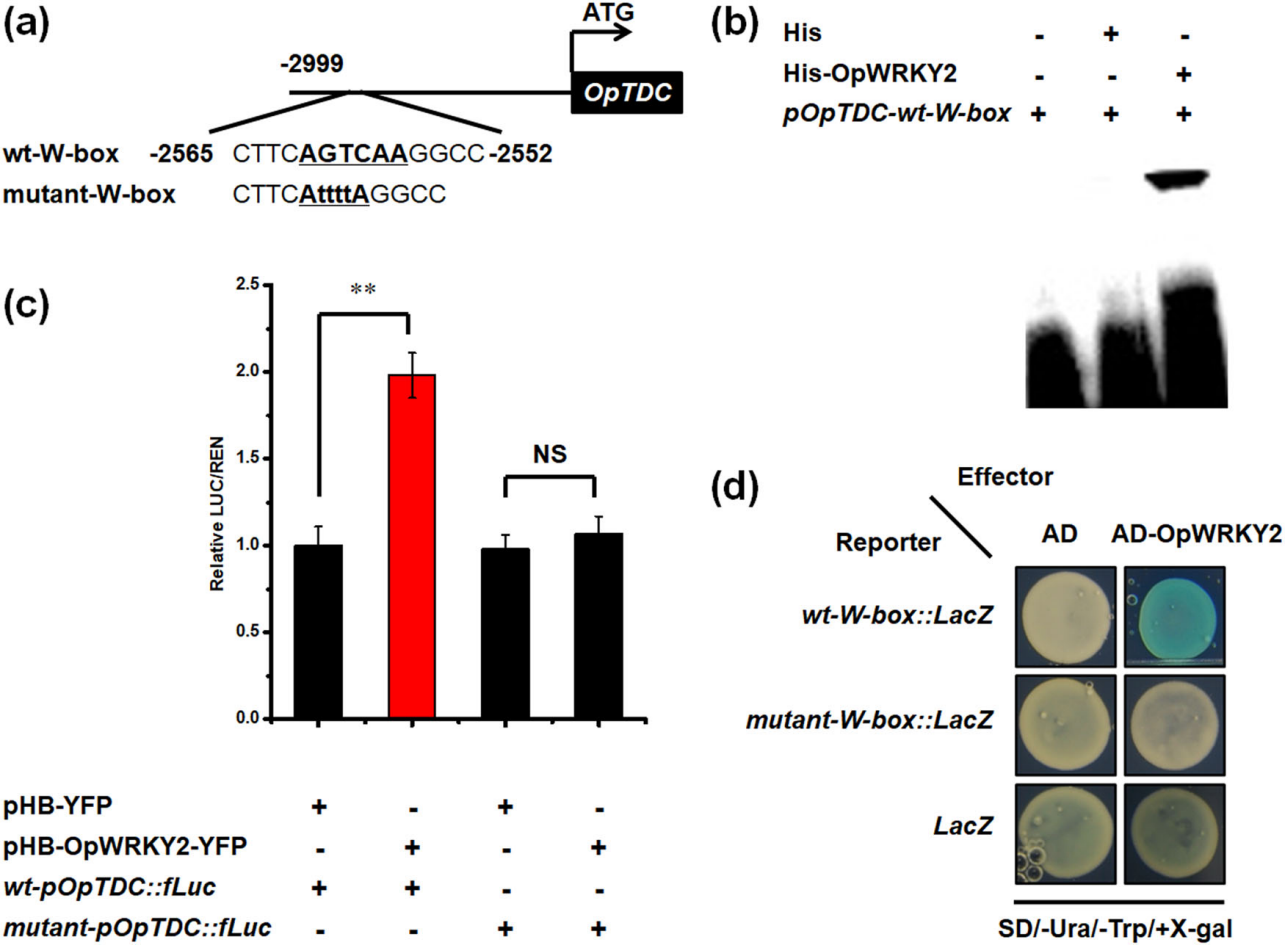
OpWRKY2 binds and activates the promoter of *OpTDC* in vitro and in vivo. **a** Diagram of the *OpTDC* promoter fragment and the sequence of a subfragment containing a W-box motif or mutant W-box motif. **b** Electrophoretic mobility shift assay (EMSA) indicating that OpWRKY2 binds to the W-box in the *OpTDC* promoter. pCold-HIS protein without OpWRKY2 was used as the control. **c** Dual-luciferase (Dual-LUC) assays showed the activation effect of OpWRKY2 on the *OpTDC* promoter. The *OpTDC* promoter and *OpTDC* promoter containing a mutant W-box motif were fused to the *firefly luciferase* reporter gene, and the promoter activity was determined by a transient Dual-LUC assay in *N. benthamiana*. The relative LUC activity was normalized to that of the reference *Renilla* (REN) luciferase. Error bars indicate the SD (*n* = 3). Student’s *t*-test: **P < 0.01; NS no significance. **d** Yeast one-hybrid (Y1H) assay indicating that OpWRKY2 binds to the W-box in the *OpTDC* promoter. Yeast cells transformed with different combinations of constructs were grown on SD/-Ura/-Trp/+X-gal medium. Pictures were taken after 4 days of incubation at 30 °C. Y1H assays were repeated three times.

To further investigate the putative role of OpWRKY2 in the regulation of *OpTDC*, the 3090 bp *OpTDC* promoter region was analyzed for potential binding sites of WRKY TFs (Fig. 7a). Indeed, a conserved W-box (TTGACT) was found in the analyzed *OpTDC* promoter region (−2561 bp to −2556 bp relative to the ATG). Next, the ability of OpWRKY2 to bind and activate the *OpTDC* promoter was analyzed in vivo and in vitro. First, the binding affinity of OpWRKY2 to the *OpTDC* promoter was determined by an EMSA. A 14 bp probe (−2565 to −2552 relative to the ATG) containing the native W-box element was used for EMSA experiments. As shown in Fig. 7b, coincubation of recombinant OpWRKY2 with the native W-box probe resulted in DNA mobility shifts, while coincubation with the control protein did not alter DNA separation. To further corroborate the binding affinity of OpWRKY2 to the W-box element in the *OpTDC* promoter, yeast one-hybrid assays were performed. OpWRKY2 fused to the yeast GAL4 activation domain served as prey, and three tandem repeats of the W-box-containing region (CTTC**AGTCAA**GGCC) of the *OpTDC* promoter (−2565 to −2552 relative to the ATG) served as bait. The mutant-W-box and an empty vector without a W-box served as the control. As shown in Fig. 7d, OpWRKY2-GAL4 was able to activate the *pOpTDC-W-box*-driven *LacZ* reporter in the Y1H system, supporting the results of the EMSA experiments (Fig. 7d).

To confirm the activation effect of OpWRKY2 on *pOpTDC* transcription, Dual-LUC assays were carried out in *N. benthamiana* leaves. The reporter constructs were obtained by inserting the 3090 bp native promoter of *OpTDC* and a mutated version with point mutations in the W-box element into the *pGreenII0800-LUC* vector. *OpWRKY2-YFP* was driven by the 35 S promoter, and the *pHB-YFP* construct without *OpWRKY2* was used as the negative control. Compared with the *pHB-YFP* control group, the activation ability in OpWRKY2-*pOpTDC* coinfiltration was significantly increased (Fig. 7c). In contrast, the reporter gene was not activated in the mutated promoter constructs (Fig. 7c). Taken together, these results suggest that OpWRKY2 positively regulates camptothecin biosynthesis by binding a W-box element in the *OpTDC* promoter and transcriptionally activating this key pathway enzyme-encoding gene.

## Discussion

### Camptothecin biosynthesis in *O. pumila* hairy roots is dynamic

Camptothecin is an important anticancer drug produced in a variety of unrelated plants. The market supply of camptothecin is currently provided by extraction from woody plants. *O. pumila*, a perennial herb, has recently been established as an alternative source of camptothecin. The hairy-root transformation system of *O. pumila* has been optimized for high camptothecin production and has been proposed as a major source of camptothecin for pharmaceutical markets^7^. The hairy-root system has a variety of features, such as rapid growth, elicitation ability, and free hormones^33,34^. To further optimize camptothecin production in *O. pumila* hairy roots, it is important to better understand the regulatory mechanism associated with camptothecin biosynthesis. However, there has been no research carried out on the dynamic changes in camptothecin biosynthesis over the cultivation period of hairy roots.

In this study, the biomass of *O. pumila* hairy roots and the total yield of camptothecin gradually increased and stabilized after 50 days. Therefore, camptothecin accumulation in *O. pumila* hairy roots reached a peak at 40 days and then began to decline, whereas the levels of secreted camptothecin increased throughout the analyzed time period and represented 40% of the total camptothecin in the culture at 60 days. In accordance with the levels of camptothecin production in hairy roots, the expression levels of camptothecin biosynthetic genes rapidly increased for the first 40 days of cultivation and then decreased to almost no expression until day 60. The most drastic changes in expression among the camptothecin biosynthetic genes were observed for *OpTDC* and *OpSTR*, with up to 5.32-fold upregulation of *OpTDC* at 40 days. *OpTDC* and *OpSTR* are central biosynthesis genes involved in the camptothecin pathway. OpTDC is considered a rate-limiting factor in CPT biosynthesis, and suppression of *OpTDC* activity results in decreased production of camptothecin in *O. pumila* hairy roots^11^. Introduction of *OpSTR* into *O. pumila* hairy roots led to a significant increase in camptothecin production compared to that in a control line^8^. In addition, co-overexpression of *STR* and *G10H* from *C. roseus* resulted in CPT biosynthesis in *O. pumila* hairy roots^7^. These analyses show that camptothecin biosynthesis in *O. pumila* hairy roots changes over time. They further highlight that separate measurements of camptothecin concentration in hairy roots and liquid media are important for understanding camptothecin accumulation in *O. pumila*.

### OpWRKY2 regulates camptothecin biosynthesis by directly activating *OpTDC*

By establishing a correlation between camptothecin production and the expression of the associated biosynthesis genes, a platform for the rapid identification of candidate transcription factors was developed. Here, the platform was focused on the WRKY TF family, one of the largest TF families involved in plant growth and development, including seed development, seed dormancy and germination, biotic stress, abiotic stress, development, senescence, and secondary metabolism^13,35^. In plants, WRKY transcription factors are divided into three groups based on the number of WRKY domains and the structure of the zinc fingers^12,13^. The WRKY group II proteins are further split into five distinct subgroups (IIa-e) according to their WRKY domains^12,13^. Recently, a number of WRKY TFs of different groups have been implicated in the regulation of plant secondary metabolites such as terpenoids, phenolic compounds, and alkaloids. In *Solanum lycopersicum*, SlWRKY73 (IIb) was shown to be involved in the activation of three monoterpene synthase genes^36^. In *A. annua*, the glandular trichome-specific WRKY transcription factor AaGSW1 (IIc) was shown to positively regulate *AaCYP71AV1* and *AaORA* expression by directly binding to W-box motifs in their promoters and acting as a positive regulator in artemisinin and dihydroartemisinin production^25^. Moreover, AaWRKY1 (III) in *A. annua* was reported to promote the transcription of *AaADS*, *AaCYP71AV1*, and *AaDBR2* and positively regulate artemisinin biosynthesis^17^. GaWRKY1 (IIa) in *Gossypium arboretum* was reported to regulate the production of sesquiterpenoid gossypol by activating the (+)-δ-cadinene synthase (CAD1) gene^37^. In *S. miltiorrhiza*, both SmWRKY1 (III) and SmWRKY2 (I) were shown to positively regulate tanshinone biosynthesis by binding to a W-box in the *SmDXR* promoter and activating the central pathway gene *SmCPS*, respectively^14,15^. In *Taxus chinensis*, TcWRKY1 (IIa) was reported to regulate the biosynthesis of the diterpenoid anticancer drug Taxol^38^. In *W. somnifera*, WsWRKY1 (III) was shown to regulate the biosynthesis of triterpenoids by binding to W-box elements in the promoters of *WsSQS* and *WsSQE*^18^. In *Arabidopsis thaliana*, AtWRKY23 (IIc) and AtWRKY44 (I) were reported to regulate the production of flavanols and proanthocyanin^39,40^. In addition, a positive regulatory role of CrWRKY1 (III) in CrTDC activity and tryptamine accumulation in transgenic *C. roseus* hairy roots was identified^16^. In *Coptis japonica*, CjWRKY1 (IIc) was shown to govern the expression of berberine biosynthesis^41^. In *O. pumila*, two WRKY TFs with regulatory roles in camptothecin biosynthesis have been identified. A negative regulatory role in *OpCPR* expression and camptothecin biosynthesis was reported for OpWRKY1 (III), and a slightly positive effect on camptothecin production was shown for OpWRKY3 (III)^5,19^. However, a core WRKY TF directly involved in the regulation of camptothecin biosynthesis has not yet been discovered.

**Fig. 8:**
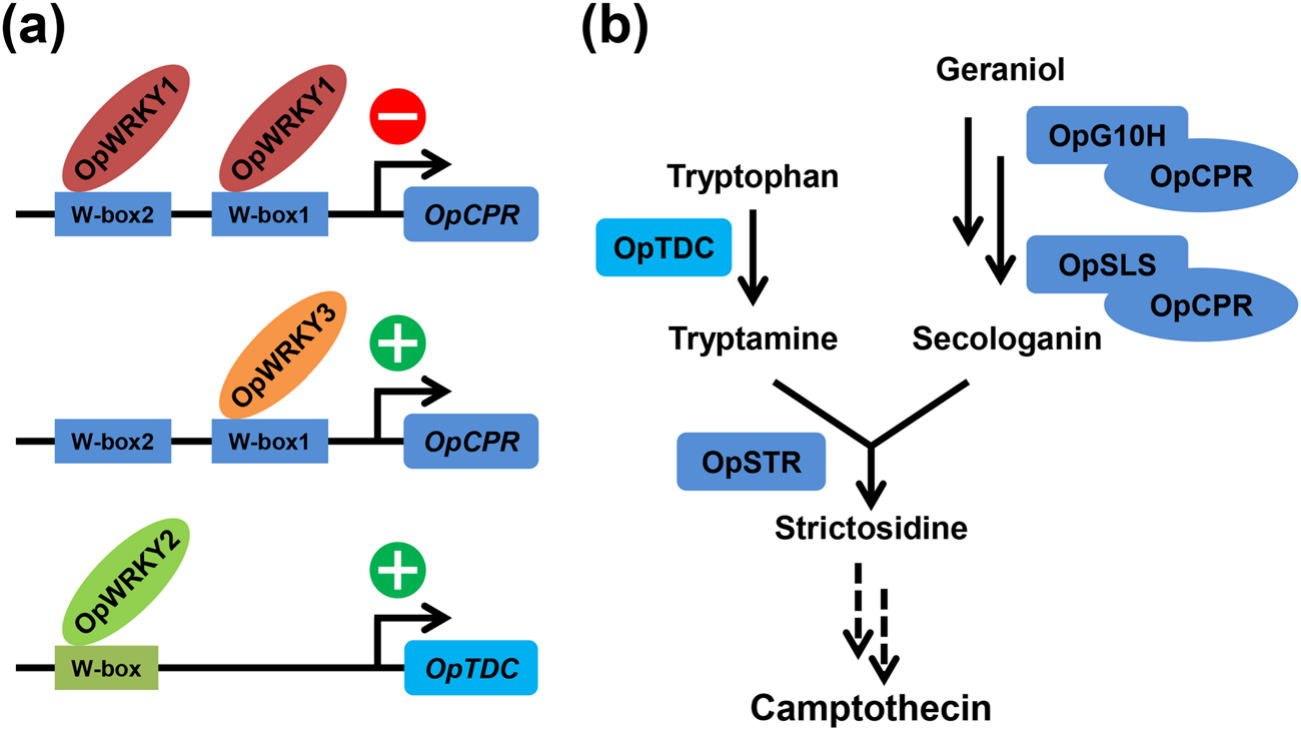
Regulatory model of camptothecin biosynthesis by OpWRKYs. **a** OpWRKY2 positively regulates camptothecin biosynthesis by binding a W-box in the *OpTDC* promoter and activating the expression of *OpTDC*. OpWRKY1 negatively regulates camptothecin biosynthesis by binding two W-boxes in the *OpCPR* promoter, while OpWRKY3 positively regulates camptothecin biosynthesis by binding one W-box in the *OpCPR* promoter. **b** Camptothecin biosynthetic pathway in *O. pumila*. Tryptophan decarboxylase (OpTDC) catalyzes the conversion of tryptophan to tryptamine. Dashed lines represent interactions that may be indirect.

Here, all putative WRKY transcription factor genes of *O. pumila* were identified, and their pattern of coexpression with the camptothecin pathway genes was analyzed. A single TF gene, OpWRKY2, showed strong transcriptional overlap with the camptothecin pathway. Overexpression of functional *OpWRKY2* resulted in increased camptothecin levels, and silencing of *OpWRKY2* correlated with decreased camptothecin levels. These results suggested a positive regulatory role of *OpWRKY2* in the camptothecin biosynthetic pathway. By detecting the expression of key enzymes of the biosynthetic pathway of camptothecin in transgenic hairy roots, it was shown that the expression of *OpTDC* was significantly upregulated in *OpWRKY2-OE* hairy-root lines. Accordingly, the expression of *OpTDC* was significantly decreased in *OpWRKY2*-silenced hairy-root lines. In *O. pumila*, *OpTDC* catalyzes the decarboxylation of tryptophan to form tryptamine, which is a key enzyme in the upstream pathway of terpenoid indole alkaloid biosynthesis^10^. EMSA, Y1H, and Dual-LUC assays showed that OpWRKY2 could bind and activate a W-box in the promoter of *OpTDC* in vitro and in vivo. In addition, the expression level of *OpCPR* was increased slightly in *OpWRKY2-OE* hairy-root lines compared to the control. Thus, the levels of loganin and secologanin, which are pathway intermediates in the iridoid branch of camptothecin biosynthesis, also showed a slight increase when *OpWRKY2* was overexpressed. Altogether, these results suggested that OpWRKY2 positively regulates camptothecin biosynthesis by binding to the W-box of *pOpTDC* and activating the expression of *OpTDC* (Fig. 8). In addition to the level of *OpWRKY2*, the expression levels of *OpWRKY3*, *OpWRKY4*, *OpWRKY5*, *OpWRKY13*, *OpWRKY15*, *OpWRKY16*, *OpWRKY21*, *OpWRKY28*, and *OpWRKY34* also showed a gradual increase before day 50. Furthermore, the expression of some *OpWRKYs*, such as *OpWRKY1*, *OpWRKY6*, and *OpWRKY9*, gradually decreased and might negatively regulate camptothecin biosynthesis. Indeed, *OpWRKY1* has been reported to negatively regulate the biosynthesis of camptothecin, while *OpWRKY3* positively regulates the biosynthesis of camptothecin^5,19^. Therefore, it is effective to screen candidate regulatory genes based on their expression levels in different growth stages, and it is necessary to continue to study the regulatory functions and mechanisms of other possible WRKY transcription factors in camptothecin biosynthesis.

Previously, a set of TFs has been implicated in the regulation of camptothecin production in *O. pumila*. Out of five ERF TF family genes (*OpERF1-5*) that had been isolated and characterized from *O. pumila* hairy roots, one TF-encoding gene, *OpERF2*, has been shown to play a positive role in regulating the iridoid biosynthesis branch of camptothecin biosynthesis^20^. Moreover, the introduction of *OpMYB1* into *O. pumila* hairy roots reduced camptothecin production and downregulated the expression level of *OpTDC*, suggesting a repressive function of *OpMYB1* in camptothecin biosynthesis^21^. In addition, two WRKY TFs with regulatory roles in *O. pumila* camptothecin biosynthesis have been identified. In contrast to the direct positive regulatory role of OpWRKY2 in *OpTDC* expression shown here, OpWRKY1 had been implicated in the suppression of camptothecin biosynthesis by binding two W-boxes in the *OpCPR* promoter and repressing the expression of *OpCPR*, while a slight involvement in camptothecin regulation has been shown for OpWRKY3, which binds via one W-box in the *OpCPR* promoter (Fig. 8)^5,19^. In addition, camptothecin accumulation was reported to be significantly increased by plant hormones such as salicylic acid and jasmonic acid in CPT-producing plants and cell cultures^42–44^. For example, camptothecin production increased under jasmonic acid treatment in cell cultures of *C. acuminata*^42^. Moreover, exogenous salicylic acid in *C. acuminata* seedlings promoted camptothecin biosynthesis^43^. In another CPT-producing plant, *Ophiorrhiza mungos*, cell suspension culture achieved significantly high camptothecin production with jasmonic acid elicitation^44^. Therefore, camptothecin accumulation is induced by hormone treatments. However, the effects of phytohormones on camptothecin accumulation in *Ophiorrhiza pumila* plants have not been reported previously, and we will further explore the effect of different plant hormones on the biosynthesis and accumulation of camptothecin in *O. pumila*.

## Conclusions

Camptothecin, a monoterpene indole alkaloid, is widely used in the treatment of cancer. This study found that camptothecin accumulation in *O. pumila* hairy roots is positively correlated with increasing culture time. Furthermore, the expression of *OpWRKY2* is correlated with camptothecin biosynthesis and induced by various phytohormones. The expression level of the camptothecin biosynthesis gene *OpTDC* was increased in the *OpWRKY2-OE* hairy-root lines and significantly downregulated in the *OpWRKY2-SRDX* hairy-root lines. Metabolite analysis in transgenic hairy roots found that *OpWRKY2* positively regulates the biosynthesis of camptothecin and tryptamine. EMSA, Y1H, and Dual-LUC assays showed that OpWRKY2 binds and activates the promoter of *OpTDC* in vitro and in vivo. Taken together, the present findings illustrate that *OpWRKY2* acts as a positive regulator in camptothecin biosynthesis and provide a feasible strategy for increasing camptothecin levels by functional WRKY proteins in *O. pumila*.

## Supplementary information

Supplementary Information
