# Supplementary material for: The transcription factor OpWRKY2 positively regulates the biosynthesis of the anticancer drug camptothecin in Ophiorrhiza pumila
Source: Hortic Res. 2021 Jan 1;8:7. doi: 10.1038/s41438-020-00437-3 (PMC7775441; doi:10.1038/s41438-020-00437-3)
Supplement: Supplementary file 1 — Supplementary Information [file 41438_2020_437_MOESM1_ESM.docx]

**Supplementary Information**

**Fig. S1.** The vector construction map. (a) Construction of the *pCAMBIA2300^+^-OpWRKY2* vector (*OpWRKY2-OE*). (b) Construction of the *pCAMBIA2300^+^-OpWRKY2-SRDX* vector (*OpWRKY2-SRDX*).


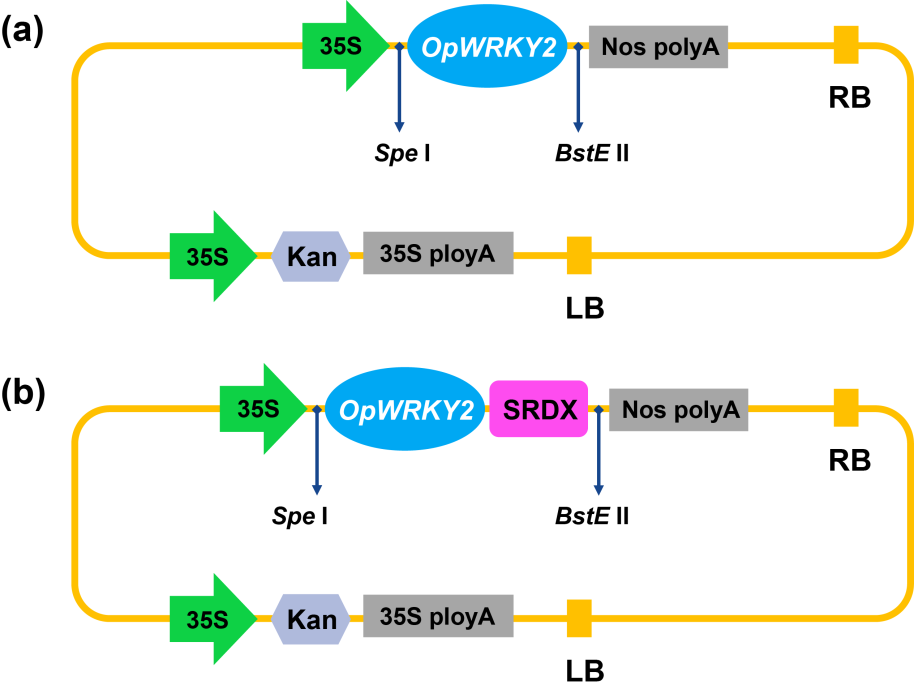


**Fig. S2.** Identification of positive *OpWRKY2-OE* transgenic hairy root lines by PCR. The *rolB* gene was used to confirm that the hairy roots were infected with *Agrobacterium*.


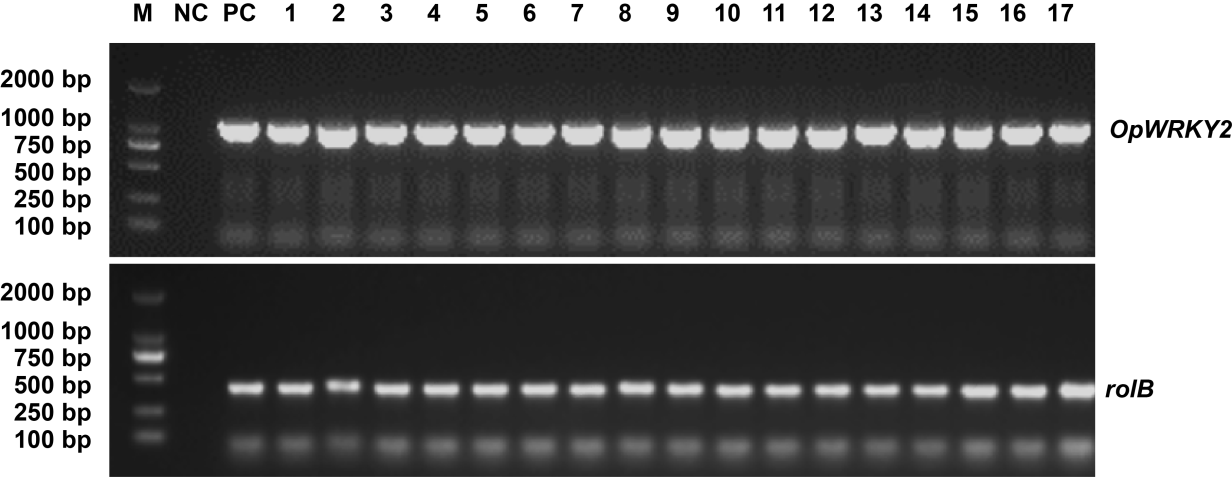


**Fig. S3.** Identification of positive *OpWRKY2-SRDX* transgenic hairy root lines by PCR. The *rolB* gene was used to confirm that the hairy roots were infected with *Agrobacterium*.


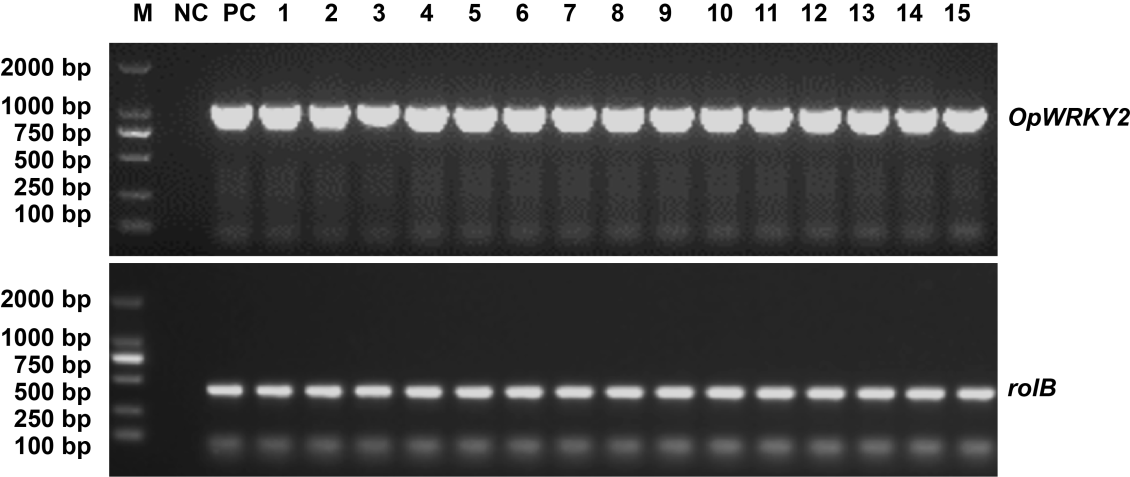


**Fig. S4.** Analysis of loganin and secologanin biosynthesis in the *OpWRKY2-OE* and *OpWRKY2-SRDX* transgenic hairy root lines. (a, b) The production of loganin (a) and secologanin (b) in *OpWRKY2-OE* and *OpWRKY2-SRDX* transgenic hairy root lines was detected by HPLC. Error bars represent the SD of three biological replicates.


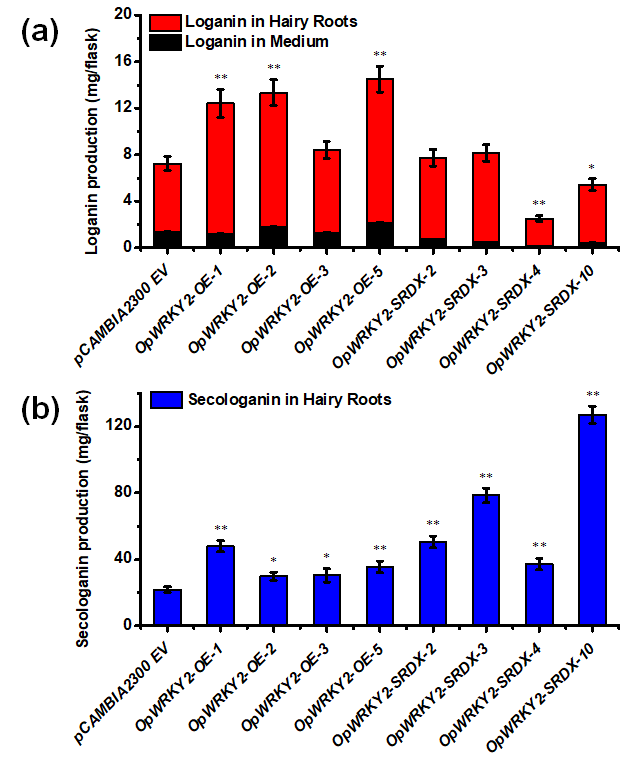


**Table S1.** Primers used for qRT-PCR experiments

| **Primers** | **Sequences (5’ to 3’)** |
| --- | --- |
| *OpWRKY1-QF_27_* | TGGACCTAACCCGCTTCCTA |
| *OpWRKY1-QR_145_* | GCAAGTTCCTCAGCAAAGCC |
| *OpWRKY2-QF_854_* | CTCCAACAACTTCGGAGTCCA |
| *OpWRKY2-QR_934_* | AGCTCTGCAGAATGTTGCCT |
| *OpWRKY3-QF_721_* | CCGACGCAACAAAATCCACA |
| *OpWRKY3-QR_901_* | TGTTATCGGCCACAGATGGG |
| *OpWRKY4-QF_45_* | TAAACCTATGCGGACTCTGGATG |
| *OpWRKY4-QR_173_* | CTTACTCGGTTCAATTCCTCTGC |
| *OpWRKY5-QF_113_* | CATCATCAGGGAAAGAAGAGCCA |
| *OpWRKY5-QR_307_* | CAGCCTCAAAGTTCCTCTTCCTT |
| *OpWRKY6-QF_365_* | ATCATAGCCCGAGTCACGAAGAACA |
| *OpWRKY6-QR_468_* | TAGTTCGCTTCCTGTAGCCTCATCG |
| *OpWRKY7-QF_566_* | AAAATTCTAAAGCCGAAAGCACA |
| *OpWRKY7-QR_708_* | TGGCTCGTCCATTTCGTTTGCTC |
| *OpWRKY8-QF_457_* | TTGGGATTCAATGTAAATACTGGTT |
| *OpWRKY8-QR_621_* | CCGATTCTCACCGTTTAGTCGCTCT |
| *OpWRKY9-QF_376_* | ATCAAGACAAAAGAAGACCAACGAT |
| *OpWRKY9-QR_579_* | TGGCTTCAAAACTTTACTCGGTGGC |
| *OpWRKY10-QF_385_* | GCTGCTACCGTGACTGAGATG |
| *OpWRKY10-QR_518_* | GTCGGCTGTATGACGGTGGAG |
| *OpWRKY11-QF_141_* | ACTATGTGAATCTGCAACTTCAAAACC |
| *OpWRKY11-QR_348_* | CTTCCTCCTTGACTTAACCTTCTTCAT |
| *OpWRKY12-QF_61_* | ATGGGAAGTAGCACCACTGGAGGGAGTA |
| *OpWRKY12-QR_252_* | AAGGGTGTTGTAATCTGATGCTGTTCC |
| *OpWRKY13-QF_124_* | GATTCTTCAGTATTACACCCTTCACAT |
| *OpWRKY13-QR_333_* | TTCACTCTGATGTTCTCCAGCCTCAAC |
| *OpWRKY14-QF_206_* | TACGCCTATTATCTTCTCAACCCACTA |
| *OpWRKY14-QR_532_* | CACTTTAGGCACCGTACTTTTCTTGTT |
| *OpWRKY15-QF_126_* | CCGATACTCATCACCAGGGACTG |
| *OpWRKY15-QR_211_* | AATTCATCTGGTTGGCAGCATAG |
| *OpWRKY16-QF_199_* | ACTTCGGATGTTAGTAATCTTTCGC |
| *OpWRKY16-QR_332_* | CTCCTTGTTTGGAAAGCGTATCTGG |
| *OpWRKY17-QF_96_* | AAACATCTTTGACATCCCTCCAT |
| *OpWRKY17-QR_233_* | TCGTAAATGGAAGGGCAAGGATA |
| *OpWRKY18-QF_4_* | GCCGTTGAACTCATAATGGGCTACAGA |
| *OpWRKY18-QR_112_* | TGAACTTCTCGACATTCTCAAGCCCAC |
| *OpWRKY19-QF_263_* | TTCCTACTGGGTCTTCAACTTTGTCTC |
| *OpWRKY19-QR_457_* | TAGAACCCACCTCACATCCCTTCATTT |
| *OpWRKY20-QF_309_* | ACAAGATTCGGGTTCTAATGTAAAAAGTG |
| *OpWRKY20-QR_427_* | GATAGTTAGGTAGTGGTGTTTGTTGGGCT |
| *OpWRKY21-QF_103_* | TCTTGTTATGATGCTCTTCTGGAGATGCC |
| *OpWRKY21-QR273* | TGAAGGTGAAGAATACGACACAGATGAGA |
| *OpWRKY22-QF25* | GAAGTAGTATCTGCCGCCGCAATCACA |
| *OpWRKY22-QR195* | CGAAGTAGAACGAGTAAATAAGCCTGA |
| *OpWRKY23-QF117* | GTGTGGCTTTGAACCTCAGCAAGATGG |
| *OpWRKY23-QR213* | ATCAAAACCACTACAACTACTGCTCTG |
| *OpWRKY24-QF33* | TCAGCAAGAGGATTCCACGAGCACC |
| *OpWRKY24-QR169* | TTGGCACCTTTACTACCTTCTTCTG |
| *OpWRKY25-QF201* | AGAAAATCATTCATTAGCCTGTGGT |
| *OpWRKY25-QR317* | ACCCTACCATCAAGATTTGGCTCCT |
| *OpWRKY26-QF139* | ATGACCCTTTTGTCTAACATCTTCTCC |
| *OpWRKY26-QR257* | TAATCAGCAGTGGCTAAAAAAGTGGGC |
| *OpWRKY27-QF393* | AACTATTCCTCCTGGTCTCAGCCCG |
| *OpWRKY27-QR519* | GTTTCGGCTACTGCCACTTGGGAGA |
| *OpWRKY28-QF13* | TCAGGAAGATTAGAACCCTCAAACT |
| *OpWRKY28-QR193* | TAAACTTAGGCACCCCAGAACTTGT |
| *OpWRKY29-QF251* | CTTCATCTCCACCTCAAACCCTA |
| *OpWRKY29-QR367* | CCTTCAACTCTTCAGCCGATTCA |
| *OpWRKY30-QF_97_* | CTTGATGGTGCCGTTACTGCCTC |
| *OpWRKY30-QR_245_* | GATGGAGAACAGACAGCAGCCCC |
| *OpWRKY31-QF_167_* | TCAAGTGGAATGGCTCAGTAGGACAAT |
| *OpWRKY31-QR_245_* | AAGTTGTTGTTCACATCCTCATTCCGA |
| *OpWRKY32-QF_17_* | TCCAGTCACGATTTCAGTCCCAACGCT |
| *OpWRKY32-QR_155_* | GCAGCATCAGAGTAGTTTCCGTTCCCA |
| *OpWRKY33-QF_17_* | TCCCGCCCGCTTCTCTACCTATCTCCC |
| *OpWRKY33-QR_367_* | AAAAGAGAACAGGCGAGTCCAAGAGCA |
| *OpWRKY34-QF_141_* | ATCCGAATCACAGCCGTCTCTAATCTA |
| *OpWRKY34-QR_367_* | GCTGGTGATGGATGATTAGTAACGGGT |
| *OpACTIN-QF_973_* | AGCAGCATGAAGATTAAGGTTGTG |
| *OpACTIN-QR_1073_* | CACATCTGCTGGAAAGTGCTG |
| *OpG10H-QF_1068_* | TGAGGAAGCCGATGTTTCCC |
| *OpG10H-QR_1252_* | TTTCATCACGCCCAATTGCC |
| *OpSLS-QF_1044_* | AAGCATCCCGAATGGCAAGA |
| *OpSLS-QR_1279_* | GAAGCATCACCGTTGGCATC |
| *OpCPR-QF_549_* | CCGGCAATATGAGCATTTCA |
| *OpCPR-QR_688_* | GTTCTCGCCAAGCAGCAAA |
| *OpTDC-QF_760_* | TAGGCTCAATCCAGGGAAAGG |
| *OpTDC-QR_907_* | TCCCACAGCAAACCTCAACA |
| *OpSTR-QF_15_* | AGCCATGGTTGTGTCGATTCT |
| *OpSTR-QR_150_* | TTCACCATCGGAGTCAAAAGC |

**TableS2.** Primers used for other experiments in this study

| **Primers** | **Sequences (5’ to 3’)** |
| --- | --- |
| *OpWRKY2-F* | ATGGAAAAGGTGAATGCTATTGAGC |
| *OpWRKY2-R* | TCAGGAGATGTATGCAATGGGGTC |
| *OpWRKY2-YFP-BamHI-F* | CTCTCTCTCAAGCTTGGATCCATGGAAAAGGTGAATGCTATTGAGC |
| *OpWRKY2-YFP-SpeI-R* | GCCCTTGCTCACCATACTAGTGGAGATGTATGCAATGGGG |
| *OpWRKY2-SpeI-F* | ACTAGTATGGAAAAGGTGAAT |
| *OpWRKY2-BstEII-R* | GGTCACCTCAGGAGATGTATGCAAT |
| *OpWRKY2-SRDX-BstEII-R* | GGTCACCTCAAGCAAAACCTAATCTAAGTTCCAGATCCAAATCCAAGGAGATGTATGCAATGGGGTC |
| *35S-F_23_* | GAGGACCTAACAGAACTCGCC |
| *rolB-F* | GCTCTTGCAGTGCTAGATTT |
| *rolB-R* | GAAGGTGCAAGCTACCTCTC |
| *pCold-OpWRKY2-BamHI-F* | TCGGTACCCTCGAGGGATCCATGGAAAAGGTGAAT |
| *pCold-OpWRKY2-HindIII-R* | GACTGCAGGTCGACAAGCTTTCAGGAGATGTATGCAAT |
| *pOpTDC-W-box-F* | CTTCAGTCAAGGCCCTTCAGTCAAGGCCCTTCAGTCAAGGCC |
| *pOpTDC-W-box-R* | GGCCTTGACTGAAGGGCCTTGACTGAAGGGCCTTGACTGAAG |
| *pGreenII 0800-pOpTDC-BamHI-F* | CAGCCCGGGGGATCCTTATTTTATATTTATTTTAGATAAT |
| *pGreenII 0800-pOpTDC-NcolI-R* | TGGCGTCTTCCATGGTCTTTAATAAAATAAGGATGATGG |
| *mutant-pOpTDC-W-box-F* | TTCTTCTTCAGTCAAGGCCAACGAG |
| *mutant-pOpTDC-W-box-R* | CTCGTTGGCCTTGACTGAAGAAGAA |
| *pLacZ-pOpTDC-W-box-EcoRI-F* | AATTCCTTCAGTCAAGGCCCTTCAGTCAAGGCCCTTCAGTCAAGGCCC |
| *pLacZ-pOpTDC-W-box-XhoI-R* | TCGAGGGCCTTGACTGAAGGGCCTTGACTGAAGGGCCTTGACTGAAGG |
| *pLacZ-mutant-pOpTDC-W-box-EcoRI-F* | AATTCCTTCATTTTAGGCCCTTCATTTTAGGCCCTTCATTTTAGGCCC |
| *pLacZ-mutant-pOpTDC-W-box-XhoI-R* | TCGAGGGCCTAAAATGAAGGGCCTAAAATGAAGGGCCTAAAATGAAGG |
| *pB42AD-OpWRKY2-EcoRI-F* | GATTATGCCTCTCCCGAATTCATGGAAAAGGTGAAT |
| *pB42AD-OpWRKY2-XhoI-R* | AGAAGTCCAAAGCTTCTCGAGTCAGGAGATGTATGCAAT |

**Table S3.** WRKY transcript factor family in *O. pumila*

| *Op*Gene ID |  | Deduced polypetide | | | WRKY group | WRKY domain | | Zinc finger domain | |
| --- | --- | --- | --- | --- | --- | --- | --- | --- | --- |
|  | CDS (bp) | ORF  (aa) | pI | MW  (Da) |  | Conserved heptapeptide | Domain number | Pattern | Zinc finger |
| *OpWRKY1* | 987 | 328 | 6.2 | 36854.4 | Ⅲ | WRKYGQK | 1 | C-X_7_-C-X_23_-HTC | C_2_HC |
| *OpWRKY2* | 1071 | 356 | 5.48 | 39808.7 | Ⅲ | WRKYGQK | 1 | C-X_7_-C-X_23_-HSC | C_2_HC |
| *OpWRKY3* | 1128 | 375 | 5.77 | 41945.1 | Ⅲ | WRKYGQK | 1 | C-X_7_-C-X_23_-HTC | C_2_HC |
| *OpWRKY4* | 945 | 314 | 8.55 | 34863.1 | Ⅱa | WRKYGQK | 1 | C-X_5_-C-X_23_-HNH | C_2_H_2_ |
| *OpWRKY5* | 918 | 305 | 6.1 | 34318.2 | Ⅱa | WRKYGQK | 1 | C-X_5_-C-X_23_-HNH | C_2_H_2_ |
| *OpWRKY6* | 1557 | 518 | 6.12 | 56187.4 | Ⅱb | WRKYGQK | 1 | C-X_5_-C-X_23_-HNH | C_2_H_2_ |
| *OpWRKY7* | 1674 | 557 | 6.55 | 60369.3 | Ⅱb | WRKYGQK | 1 | C-X_5_-C-X_23_-HNH | C_2_H_2_ |
| *OpWRKY8* | 1938 | 645 | 6.15 | 70540.9 | Ⅱb | WRKYGQK | 1 | C-X_5_-C-X_23_-HNH | C_2_H_2_ |
| *OpWRKY9* | 1779 | 555 | 6.07 | 59683.2 | Ⅱb | WRKYGQK | 1 | C-X_5_-C-X_23_-HNH | C_2_H_2_ |
| *OpWRKY10* | 1035 | 344 | 5.45 | 37556.4 | Ⅱc | WRKYGQK | 1 | C-X_4_-C-X_23_-HCH | C_2_H_2_ |
| *OpWRKY11* | 669 | 222 | 8.79 | 25521.6 | Ⅱc | WRKYGQK | 1 | C-X_4_-C-X_23_-HIH | C_2_H_2_ |
| *OpWRKY12* | 1056 | 351 | 6.36 | 39162.6 | Ⅱc | WRKYGQK | 1 | C-X_4_-C-X_23_-HNH | C_2_H_2_ |
| *OpWRKY13* | 978 | 325 | 6.57 | 36557.7 | Ⅱc | WRKYGQK | 1 | C-X_4_-C-X_23_-HNH | C_2_H_2_ |
| *OpWRKY14* | 861 | 286 | 9.38 | 32044.2 | Ⅱc | WRKYGQK | 1 | C-X_4_-C-X_23_-HNH | C_2_H_2_ |
| *OpWRKY15* | 660 | 219 | 6.07 | 25122.8 | Ⅱc | WRKYGKK | 1 | C-X_4_-C-X_23_-HNH | C_2_H_2_ |
| *OpWRKY16* | 588 | 195 | 9.24 | 21841.3 | Ⅱc | WRKYGQK | 1 | C-X_4_-C-X_23_-HSH | C_2_H_2_ |
| *OpWRKY17* | 1140 | 379 | 5.87 | 41537.4 | Ⅱc | WRKYGQK | 1 | C-X_4_-C-X_23_-HIH | C_2_H_2_ |
| *OpWRKY18* | 1059 | 352 | 9.2 | 38726.9 | Ⅱd | WRKYGQK | 1 | C-X_5_-C-X_23_-HNH | C_2_H_2_ |
| *OpWRKY19* | 1026 | 341 | 9.66 | 36508.5 | Ⅱd | WRKYGQK | 1 | C-X_5_-C-X_23_-HRH | C_2_H_2_ |
| *OpWRKY20* | 1053 | 350 | 9.54 | 39482.7 | Ⅱd | WRKYGQK | 1 | C-X_5_-C-X_23_-HNH | C_2_H_2_ |
| *OpWRKY21* | 1044 | 277 | 8.39 | 31088.6 | Ⅱe | WRKYGQK | 1 | C-X_5_-C-X_23_-HNH | C_2_H_2_ |
| *OpWRKY22* | 1323 | 440 | 5.43 | 48173.3 | Ⅱe | WRKYGQK | 1 | C-X_5_-C-X_23_-HNH | C_2_H_2_ |
| *OpWRKY23* | 1065 | 354 | 5.92 | 38564.5 | Ⅱe | WRKYGQK | 1 | C-X_5_-C-X_23_-HNH | C_2_H_2_ |
| *OpWRKY24* | 480 | 159 | 9.03 | 17447.2 | Ⅱe | WRKYGQK | 1 | C-X_5_-C-X_23_-HNH | C_2_H_2_ |
| *OpWRKY25* | 1662 | 553 | 6.62 | 60761.9 | Ⅰ | WRKYGQK/WRKYGQK | 2 | C-X_4_-C-X_22_-HEH / C-X_4_-C-X_22_-HDH | C_2_H_2_ / C_2_H_2_ |
| *OpWRKY26* | 1584 | 527 | 8.07 | 57247 | Ⅰ | WRKYGQK/WRKYGQK | 2 | C-X_4_-C-X_22_-HNH / C-X_4_-C-X_22_-HNH | C_2_H_2_ / C_2_H_2_ |
| *OpWRKY27* | 2211 | 736 | 6.01 | 79089.2 | Ⅰ | WRKYGQK/WRKYGQK | 2 | C-X_4_-C-X_22_-HNH / C-X_4_-C-X_22_-HNH | C_2_H_2_ / C_2_H_2_ |
| *OpWRKY28* | 1680 | 559 | 8.71 | 61481.7 | Ⅰ | WRKYGQK/WRKYGQK | 2 | C-X_4_-C-X_22_-HNH / C-X_4_-C-X_22_-HNH | C_2_H_2_ / C_2_H_2_ |
| *OpWRKY29* | 1587 | 528 | 5.6 | 57480.4 | Ⅰ | WRKYGQK/WRKYGQK | 2 | C-X_4_-C-X_22_-HSH / C-X_4_-C-X_22_-HDH | C_2_H_2_ / C_2_H_2_ |
| *OpWRKY30* | 1344 | 447 | 8.73 | 49527.8 | Ⅰ | WRKYGQK/WRKYGQK | 2 | C-X_4_-C-X_22_-HNH / C-X_4_-C-X_22_-HNH | C_2_H_2_ / C_2_H_2_ |
| *OpWRKY31* | 1077 | 358 | 5.63 | 40162.4 | Ⅲ | WRKYGQK | 1 | C-X_7_-C-X_23_-HAC | C_2_HC |
| *OpWRKY32* | 1860 | 619 | 6.43 | 67206.8 | Ⅰ | WRKYGQK/WRKYGQK | 2 | C-X_4_-C-X_22_-HDH / C-X_4_-C-X_22_-HNH | C_2_H_2_ / C_2_H_2_ |
| *OpWRKY33* | 1752 | 583 | 8.39 | 64355.5 | Ⅰ | WRKYGQK/WRKYGQK | 2 | C-X_4_-C-X_22_-HNH / C-X_4_-C-X_22_-HNH | C_2_H_2_ / C_2_H_2_ |
| *OpWRKY34* | 1092 | 363 | 9.48 | 39742.9 | Ⅱd | WRKYGQK | 1 | C-X_5_-C-X_22_-HNH | C_2_H_2_ |
